# Prenatal Alcohol Exposure, Anesthesia, and Fetal Loss in Baboon Model of Pregnancy

**Published:** 2018-06

**Authors:** Kelsey North, Ana Tobiasz, Ryan D. Sullivan, Zoran Bursac, Jose Duncan, J. Pierce Sullivan, Steven Davison, Danielle L. Tate, Stacey Barnett, Giancarlo Mari, Anna N. Bukiya

**Affiliations:** 1 Department of Pharmacology, University of Tennessee Health Science Center, Memphis, TN 38163, USA; 2 Department of Obstetrics and Gynecology, University of Tennessee Health Science Center, Memphis, TN 38163, USA; 3 Department of Comparative Medicine, University of Tennessee Health Science Center, Memphis, TN 38163, USA; 4 Department of Preventive Medicine, University of Tennessee Health Science Center, Memphis, TN 38163, USA

**Keywords:** anesthesia during pregnancy, alcohol exposure in utero, fetal alcohol exposure, nonhuman primate, adverse pregnancy outcome

## Abstract

Approximately half of pregnant women engage in alcohol consumption some time during pregnancy. On the other hand, a small percentage of pregnant women undergo surgery and anesthesia at some time during pregnancy. In emergencies, anesthesia has to be administered to patients who are under alcohol intoxication. Anesthetic management during pregnancy while patients are intoxicated with alcohol is challenging. Here, we utilized a retrospective analysis of data available from 17 pregnant baboons that underwent anesthesia with alcohol exposure during mid-pregnancy. The analysis was designed to answer three questions: whether maternal vital signs remained stable under anesthesia combined with alcohol, whether maternal vital signs that were routinely monitored under anesthesia could serve as predictor(s) of fetal loss, and what the impact of the combined application of anesthesia and alcohol was on fetal loss. For the purpose of this retrospective analysis, we utilized vital sign (heart and respiratory rates, temperature, oxygen, carbon dioxide, systolic and diastolic blood pressure) and pregnancy outcome (miscarriage versus fetal survival through second trimester-equivalent of human pregnancy) records from 17 pregnant baboons that underwent gastric infusion of either control or alcohol-containing drink under isoflurane anesthesia during the second trimester-equivalent of human pregnancy. Half of the dams underwent a brief prior anesthetic episode for the purpose of gestational age confirmation. Thus, in our analysis, baboons were divided into four groups: “Control” without prior anesthesia, “Control” with prior anesthesia, “Alcohol” without prior anesthesia, and “Alcohol” with prior anesthesia. We did not detect any maternal vital sign in any of the groups that would be predictive of a fetal loss. However, prior anesthesia predisposed dams to the risk of lowering maternal systolic blood pressure and to a significant decrease in maternal oxygen level during the combined application of anesthesia and alcohol. Conceivably, our data showed the largest fetal loss in this group. The disruptive nature of anesthesia and alcohol on maternal vital parameters warns against the use of anesthesia in combination with alcohol during pregnancy.

## 1. Introduction

International studies report that 5%–60% of pregnant women engage in alcohol consumption at some time during pregnancy [1, 2, 3, 4, 5]. As documented by numerous studies, binge alcohol consumption with sharp peaks for blood alcohol concentration (BAC) above 80 mg/dL produces the most negative effects on the fetus, including development of fetal alcohol syndrome and fetal alcohol spectrum disorders (FASD) [6, 7, 8]. Moreover, higher amounts of blood alcohol were found to be associated with increased risk of pregnancy loss [9].

It is estimated that 0.5%–2% of all pregnant women undergo surgery and anesthesia at some time during pregnancy [10, 11, 12]. Although an isolated episode of anesthesia generally does not pose teratogenic risk to the fetus [10, 11], anesthesia during the first trimester increases the risk of spontaneous abortion [11]. Yet, there is no readily accessible physiological predictor of fetal loss in response to anesthesia. Anesthetic management in patients under alcohol intoxication represents additional challenges [13]. Indeed, alcohol interacts with a variety of commonly used anesthetics, and modifies their effect on key physiological characteristics, including cardiac and neuronal function [14, 15, 13, 16, 17]. Although alcohol in combination with anesthesia during pregnancy represents a highly rare case, these cases take place in emergency settings. The effect of such combinations on maternal vital signs and pregnancy outcome remains unknown.

In the present study, we used a retrospective analysis of pregnancy outcome records and vital sign monitoring during anesthesia from pregnant baboons that underwent anesthesia during a study focusing on the consequences of prenatal alcohol exposure to the fetal cerebral artery [18, 19]. Baboons were first sedated with ketamine, then deep anesthesia was achieved and maintained with isoflurane. These compounds are on the World Health Organization’s List of Essential Medicines (http://www.who.int/medicines/publications/essentialmedicines/en/) and used in operative settings as injectable and inhalable anesthetics, respectively [20, 21, 22]. Their use includes surgical interventions during pregnancy [23, 24]. The analysis was aimed to answer three questions: (1) whether maternal vital signs remained stable under anesthesia combined with alcohol, (2) whether maternal vital signs that were routinely monitored under anesthesia could serve as predictor(s) of fetal loss, and (3) what impact a combined application of anesthesia and alcohol had on fetal loss.

## 2. Materials and methods

### 2.1. Ethics and study approval

The care of animals and experimental protocols were reviewed and approved by the Institutional Animal Care and Use Committee of the University of Tennessee Health Science Center, which is an AAALAC International-accredited institution.

### 2.2. Animal subjects

Baboons were received from the Primate Research Center, University of Oklahoma, over the course of 2.5 years (2014–2017). The study involved *Papio hamadryas anubis* dams (ages 7–15 y) carrying single female fetuses. The gestational age of candidate baboons for the study was determined by the center based on visual observations of female baboon cycles. In addition, in half of the cases additional confirmation of fetal gestational age was achieved with Doppler ultrasound examination under ketamine (10 mg/kg of body weight) anesthesia that lasted 15–20 min. Before reaching 80 d of gestation, baboon dams were transported to the University of Tennessee Health Science Center (UTHSC) and given ten days to acclimate to the new environment. Dams were alcohol-na¨ıve but had been used in other research studies. At UTHSC, dams were single-housed in standard baboon cages, with visual and audio access to each other. A maximum of four baboons in cages were housed per room, on a 12-hour light/dark cycle (lights on at 6:00 AM). Baboons were fed twice a day, each feeding consisting of the High Protein Monkey Diet (~15 biscuits per meal, 21 kcal/biscuit) to sustain a baboon’s weight gain as expected throughout the pregnancy. Each feeding was also supplemented by two servings of fresh fruit or vegetables and two tablespoons of peanut butter. Drinking water was available ad libitum. The facilities were maintained in accordance with the USDA and the Association for Assessment and Accreditation of Laboratory Animal Care standards. Species-appropriate toys and video presentations were provided to dams on a daily basis as means of environmental and social enrichment. Housing conditions were consistent throughout the experimental procedures.

### 2.3. Confirmation of gestational age and drink infusion procedures

Fetal outcome and maternal vital sign under anesthesia records were available from baboons that underwent anesthesia during a study on the consequences of prenatal alcohol exposure to the fetal cerebral artery as described [18, 19]. In particular, dams were subjected to either alcohol or control infusion procedures at 90 d, 100 d, and 110 d of gestation (dGA). On the day of the first procedure (at 90 ± 5 dGA, term at 175 dGA), animals were anesthetized and gestational age was confirmed by Doppler sonography using SonoAce R3 by Samsung (Seoul, South Korea) or GE Voluson E series sonographer (General Electric Company, Fairfield, CT, USA). Dams were randomly assigned to the four study groups described in Section 2.5. For the two “Alcohol” groups, the infusion contained 1.8 g/kg ethanol (ultrapure, 200 proof; American Bioanalytical, Natick, MA, USA) diluted in reverse osmosis purified drinking water. This alcohol dose rendered 80 mg/dL alcohol on average in maternal blood and 63 mg/dL alcohol in amniotic fluid [18]. The two “Control” groups of animals received an isocaloric solution containing orange-flavored Tang powder (Kraft Foods; Northfield, IL, USA). In both cases, the total volume of the infusion solution was 200 mL. Prior to each infusion procedure, the animals were fasted for 12 h. On the day of the infusion, the animal sedation was induced by a single shot of ketamine hydrochloride (Ketaset, 10 mg/kg of body weight). Throughout the infusion, anesthesia was maintained with isoflurane. Animals were provided with fluid support (75 mL/h, 0.9% NaCl) throughout anesthesia. A rectal suppository of indomethacin (25 mg) was used to prevent labor during the procedure. A gastric catheter was introduced into the stomach and the infusion was administered over 10 min. During each infusion episode, animals remained under isoflurane anesthesia for up to 200 min. Both groups of animals received a single IM injection of carprofen (Rimadyl, 4.4 mg/kg of body weight) to alleviate symptoms of hangover.

### 2.4. Outcomes

The percentage of administered isoflurane (%) was recorded every 10 min throughout each infusion episode. Maternal vital signs were monitored continuously using SurgiVet Advisor Vital Signs Monitor V9204 (Smiths Medical, Waukesha, WI, USA) and standard supplies. Maternal heart rate (beats/min), respiratory rate (breaths/min), peripheral capillary oxygen saturation (SpO_2_, %), end-tidal carbon dioxide (ETCO_2_, mmHg), systolic and diastolic blood pressure (mmHg), and temperature (°C) were recorded every 10 min throughout each infusion episode. Fetal survival was monitored throughout 120 dGA.

### 2.5. Data analysis

**Figure 1:**
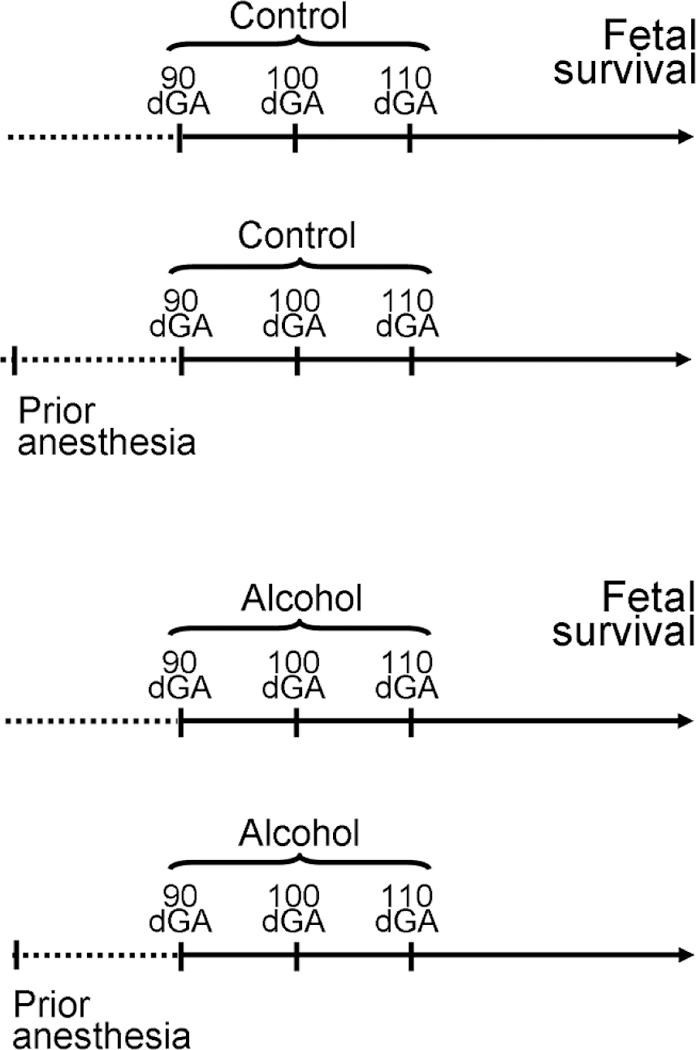
Schematic chart outlining experimental design. For the purpose of this retrospective study, we utilized vital sign and pregnancy outcome records from 17 baboons: 5 baboons in “Control” group without prior anesthesia (Control), 3 baboons in “Control” group with prior anesthesia (Control-PA), 5 baboons in “Alcohol” group without prior anesthesia (Alcohol), and 4 baboons in “Alcohol” group with prior anesthesia (Alcohol-PA). On gestational days 90, 100, and 110, pregnant dams received gastric infusion of either control or alcohol-containing drink. Fetal survival was monitored throughout 120 dGA.

For the purpose of this retrospective analysis, we utilized vital sign and pregnancy outcome records from 17 baboons: 5 baboons in the “Control” group without prior anesthesia (Control), 3 baboons in the “Control” group with prior anesthesia (Control-PA), 5 baboons in the “Alcohol” group without prior anesthesia (Alcohol), and 4 baboons in the “Alcohol” group with prior anesthesia (Alcohol-PA) (Figure 1). Vital sign (outcome) measurements included in the statistical analysis corresponded to 100 min throughout 200 min time interval following the start of isoflurane anesthesia during the first infusion episode at 90 dGa. At 100 min into isoflurane anesthesia during this procedure, a level of 80 mg/dL alcohol was reached in maternal blood for the alcohol-exposed baboons and remained steady throughout 100–200 min as reported in [18]. We did not pursue the analysis during treatments 2 and 3, as fetal losses occurred after treatment 1.

Final plotting of the data was performed using Origin 8.5.1 (OriginLab; Northampton, MA, USA). Fitting and statistical analyses of data were conducted using SAS/STAT 14.1 (SAS Institute Inc., Cary, NC, USA). In order to test the effects of group exposure, time, and group × time interaction on the outcome differences, we applied a multivariable linear regression model with repeated measures over time, with the assumption of a compound symmetry covariance structure. We further tested the outcome differences between dead fetuses and those that lived to the end of the study by adding fetal loss covariate to the model. In order to test the fetal loss rate differences between the groups, we applied Fishers Exact test and Exact Chi-square test. Associations were considered significant at the alpha level of 0.05.

## 3. Results

### 3.1. Maternal vital signs

**Figure 2:**
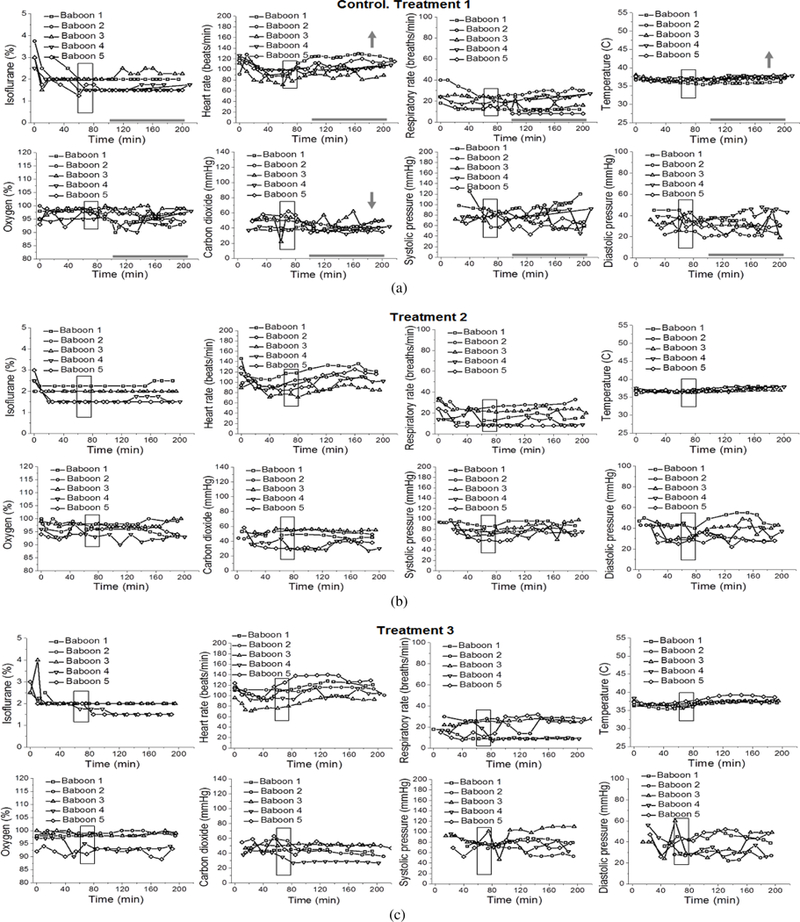
Maternal vital signs during each episode of gastric infusion under anesthesia in “Control” group without prior anesthesia (Control). Here and in Figure 3, treatments 1, 2, and 3 correspond to gastric infusion of control (isocaloric to alcohol) solution at 90 dGa, 100 dGa, and 110 dGa, respectively. Maternal heart rate (beats/min), respiratory rate (breaths/min), peripheral capillary oxygen saturation (%), end-tidal carbon dioxide (mmHg), systolic and diastolic blood pressure (mmHg), and temperature (°C) were recorded every 10 min throughout each infusion episode. Here, and in Figures 3–5, boxed areas correspond to the time of drink infusion. Bold grey horizontal lines highlight the anesthesia time interval during which maternal vital signs were analyzed for the purpose of this retrospective study. This interval corresponds to the timing of steady maternal BAC at ≈80 mg/dL [18]. Grey arrows indicate direction of the statistically significant change in the measured parameter over the course (100–200 min) of anesthesia during treatment 1 within each group. We did not pursue the analysis during treatments 2 and 3, as fetal losses occurred after treatment 1 (Figures 3–5). For differences between the groups, please refer to Table 1. For details of statistical analysis, please refer to Section 2.5.

**Figure 3:**
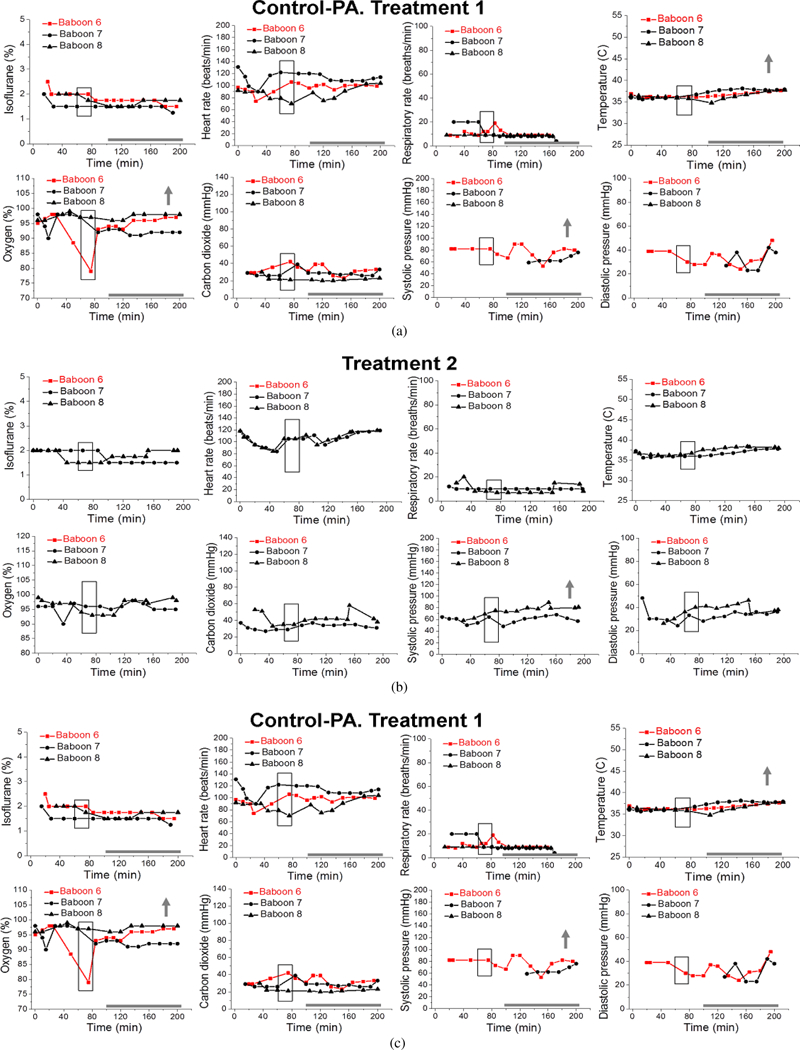
Maternal vital signs during each episode of gastric infusion under anesthesia in “Control” group with prior anesthesia (Control-PA). Annotations are as in Figure 2. Red symbols and lines represent vital signs of a pregnant dam which had her pregnancy ended with a miscarriage after treatment 1.

**Figure 4:**
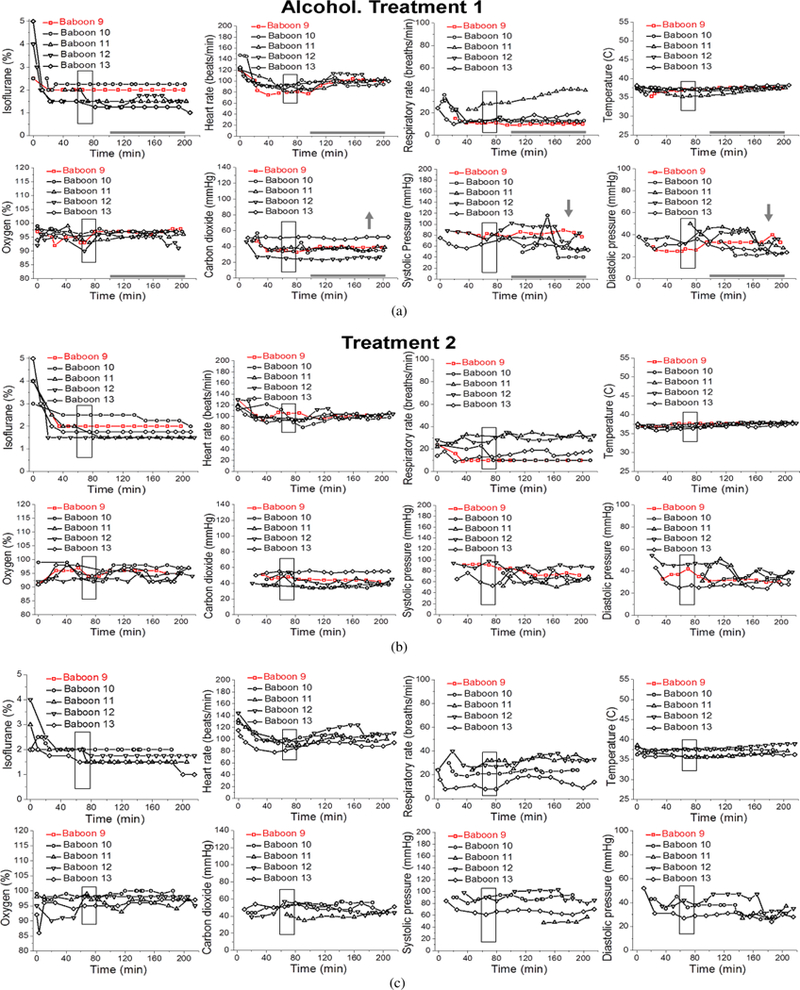
Maternal vital signs during each episode of gastric infusion under anesthesia in “Alcohol” group without prior anesthesia (Alcohol). Here and in Figure 5, treatments 1, 2, and 3 correspond to gastric infusion of alcohol-containing drink (1.8 g/kg) at 90 dGa, 100 dGa, and 110 dGa, respectively. Maternal heart rate (beats/min), respiratory rate (breaths/min), peripheral capillary oxygen saturation (%), end-tidal carbon dioxide (mmHg), systolic and diastolic pressure (mmHg), and temperature (°C) were recorded every 10 min throughout each infusion episode. Red symbols and lines represent vital signs of a pregnant dam which had her pregnancy ended with a miscarriage after treatment 2.

**Figure 5:**
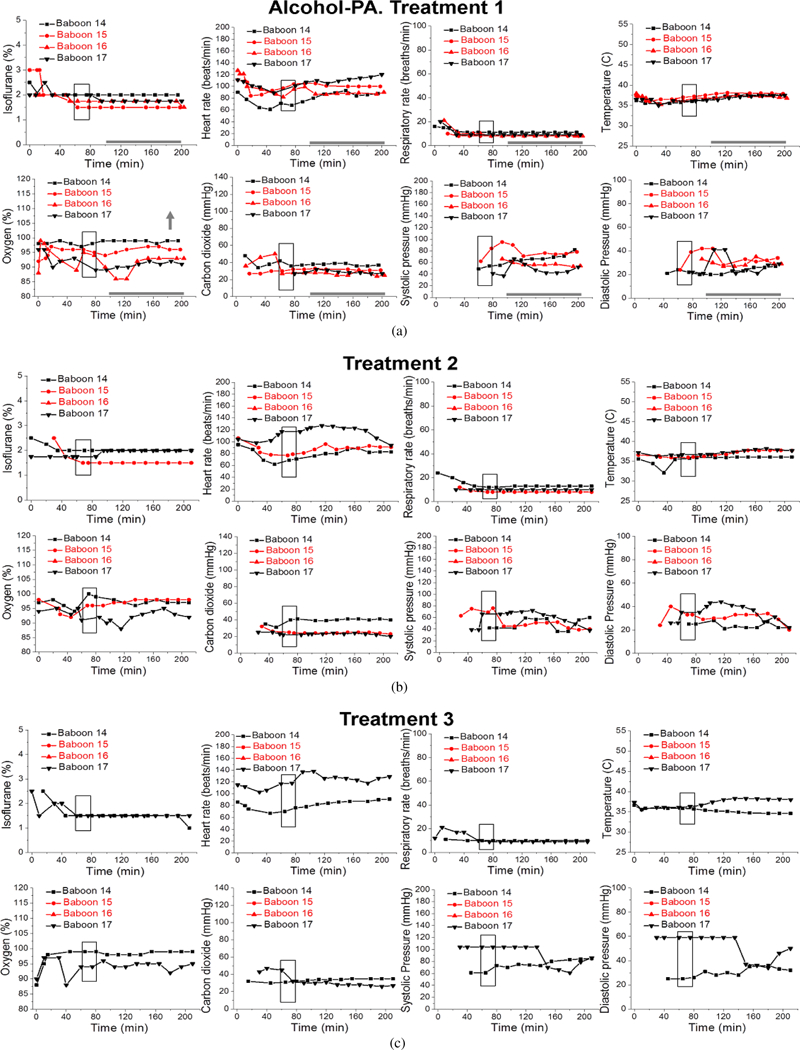
Maternal vital signs during each episode of gastric infusion under anesthesia in “Alcohol” group with prior anesthesia (Alcohol-PA). Annotations are as in Figure 4. Red symbols and lines represent vital signs of two pregnant dams which had their pregnancies ended with miscarriages after treatments 1 and 2.

Statistical analysis was performed on data from the first infusion episode (treatment 1) of Control, Control-PA, Alcohol, and Alcohol-PA groups (Figures 2–5).

Isoflurane levels to maintain deep anesthesia at 100–200 min did not differ between groups, and remained constant at ~ 1.7% throughout the measured interval between 100 min and 200 min (Figures 2–5; Table 1). Thus, we proceeded to analysis of the maternal vital signs during this time interval.

Maternal heart rate in all four groups significantly increased with the passage of time (from 100 min to 200 min) that dams spent under anesthesia (*P* = .0160). However, within individual groups, maternal heart rate was only significantly increased over time within the Control group (Figure 2(a)). Average maternal heart rate did not differ significantly between the Control and three other groups.

Maternal respiratory rate did not show statistically significant changes over time or between the groups.

Maternal temperature in all four groups significantly increased from the baseline average with the passage of time (from 100 min to 200 min) that dams spent under anesthesia (*P* <.0001). In absolute values, such increase was modest, reaching 0.83 °C at maximum. Within individual groups, Control and Control-PA rendered significantly larger increases in temperature over the course of anesthesia when compared to the remaining groups (*P* = .0326) (Figures 2(a) and 3(a)). However, average maternal temperature did not differ significantly between the Control and three other groups due to variations in the average temperature at the beginning of each group’s time interval under analysis as following: 36.6 °C (Control), 36.2 °C (Control-PA), 36.7 °C (Alcohol), 36.7 °C (Alcohol-PA).

Overall, maternal oxygen significantly increased with the passage of time (from 100 min to 200 min) that dams spent under anesthesia (*P* = .0190). These increases were mostly apparent for the Control-PA and Alcohol-PA groups (Figures 3(a) and 5(a)); yet, they did not differ significantly from the other groups. Average maternal oxygen level in the Alcohol-PA group was significantly lower when compared to Control (Table 1). In addition, oxygen in the Alcohol-PA group was significantly less than in the Control-PA group (*P* =.0382).

Maternal carbon dioxide significantly changed with the passage of time (from 100 min to 200 min) that dams spent under anesthesia (*P* = .0117). In the Control group, carbon dioxide level significantly declined over the course of anesthesia (Figure 2(a)), while in the Alcohol group carbon dioxide level showed a significant increase over the course of anesthesia (*P* = .0169) (Figure 4(a)). Average carbon dioxide levels in the Control-PA, Alcohol, and Alcohol-PA groups were significantly lower when compared to the Control group (Table 1).

Maternal systolic blood pressure remained relatively unchanged throughout the duration of anesthesia, although a transient significant increase was detected in the Control-PA group (Figure 3(a)), while a transient significant decrease was present in the Alcohol group (Figure 4(a)). Average maternal systolic blood pressure in the Alcohol-PA group was 21.22 mmHg lower than in the Control group (Table 1). Maternal diastolic blood pressure in all four groups significantly declined with the passage of time (from 100 min to 200 min) that dams spent under anesthesia (*P* = .0013). Within individual groups, the decline was statistically significant only within the Alcohol group (Figure 4(a)). Average maternal diastolic pressure did not differ significantly between the Control and three other groups.

With regard to successful pregnancies and pregnancies ending in fetal loss, no differences were detected in any of the measured maternal parameters during anesthesia.

### 3.2. Fetal loss

**Figure 6:**
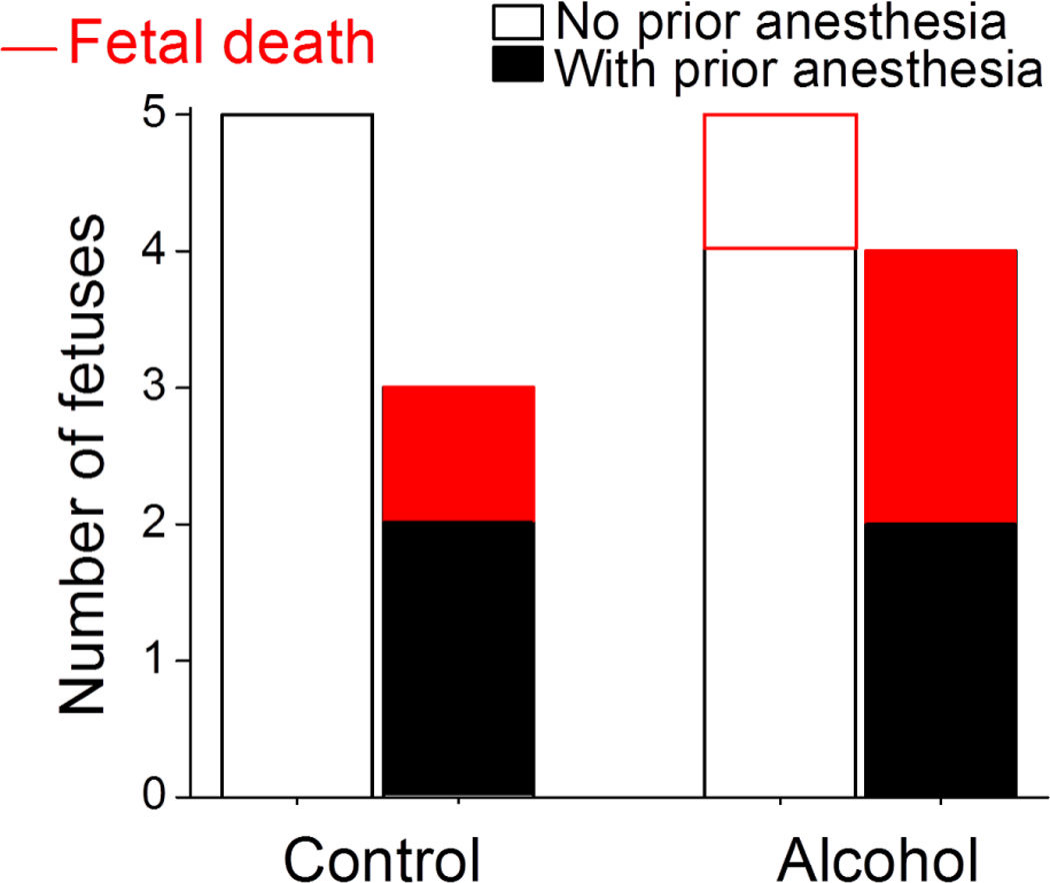
Bar graph summarizing number of lost (red) and survived fetuses (black) in each of the four experimental groups. Each bar represents number of fetuses/pregnant dams in corresponding group. Red-shaded portions of the bars reflect number of pregnancies that ended with a miscarriage. Largest fetal loss (2 out of 4) occurred in Alcohol group with prior anesthesia (Alcohol-PA, last bar).

In the Control group, there were no fetal losses during the study (Figure 6). In the Control-PA and Alcohol groups, one fetus was lost per group, rendering 33% and 20% fetal loss, respectively. In the Alcohol-PA group, however, two pregnancies out of four were lost, a 50% fetal loss (Figure 6). Comparison of fetal loss data from the Control group and Alcohol-PA group did not yield formal statistical significance (*P* =.1667, Fisher’s exact test). However, considering the relatively low *P*-value, we performed post-hoc power analysis. It was estimated that we would need 10 animals in each group instead of 4–5 to detect a 50% difference in fetal loss rate at the alpha level of 0.05.

## 4. Discussion

In the present study, we analyzed maternal vital signs under conditions of both anesthesia and alcohol treatment in baboons for the first time. Although vital signs in the four groups under analysis exhibited features that were unique to a particular group, we did not detect any maternal vital signs that would be predictive of a fetal loss. However, alcohol with repeated anesthesia poses a risk of lowering maternal systolic blood pressure and significantly lowers maternal oxygen level. Moreover, our data showed the largest rate of fetal loss (50%) in the group treated with an alcohol infusion coupled with prior anesthesia.

The current study utilized pregnant baboons. The difficulty of studying alcohol exposure during pregnancy in humans arises from two interrelated limitations. First, human subjects greatly differ in their habits of alcohol consumption. Thus, standardization of drinking patterns and amounts of consumed alcohol is an obstacle when interpreting the alcohol-related outcomes. Second, prenatal alcohol exposure results in a wide range of developmental abnormalities (i.e., FASD) representing the leading preventable cause of mental retardation in the western world [25, 26, 27, 28]. Thus, intended exposure of pregnant women to alcohol is unfeasible for ethical reasons. In this regard, standardized alcohol exposure during timed pregnancy in animal models has been widely used as an alternative [29, 30, 31]. Nonhuman primates (including baboons) are often utilized in various studies that involve anesthesia [32, 33, 34] or alcohol consumption [35, 36], including studies on anesthesia or alcohol exposure during pregnancy [18, 19, 22, 37]. Appropriately for this study, baboon reproductive physiology is similar to humans [38]. Moreover, the hormonal control of pregnancy and causes of pregnancy loss were found to be similar in baboon and human populations [39, 40, 41]. Although animal models have apparent limitations (such as differences in genetic background when compared to humans), findings in nonhuman primate models of pregnancy have high translational potential. In our study, pregnancy loss occurred between 90 d and 110 d of baboon pregnancy (red symbols and lines in Figures 3–5) which is within the second trimester equivalent of human pregnancy (baboon term is approximately 175 ± 11 dGa; [40, 42]). Notably, this interval is outside of the high-risk time periods described for baboon fetal loss: prior to 90, and during 175–180 days of gestation [40]. Moreover, while the natural rate of miscarriage in baboons is about 15% [40], average fetal loss for all four groups was 24% during the course of this study. Therefore, pregnancy loss in our study did not follow the natural pregnancy loss frequency for baboons, and therefore could be the result of an environmental insult.

In our analysis, we used data obtained from baboons that underwent alcohol exposure episodes resulting in maternal BAC of ≈80 mg/dL (≈17 mM), according to the timing of steady maternal BAC described by Seleverstov et al. [18]. This concentration represents the legal limit of alcohol intoxication for driving a motor vehicle in most of the United States. Considering that lethal BAC in humans averages 355 mg/dL [43], BAC in our study constitutes a rather modest and clinically relevant amount.

During alcohol infusion, pregnant dams were under anesthesia. This condition lasted for a total of 180–200 min to allow for maternal blood collections for the measurement of alcohol levels as reported in our earlier work [18]. For the purpose of the current analysis, baboons were divided into four groups according to infusions of control versus alcohol-containing drink and absence versus presence of prior short anesthetic episodes; the previous anesthesia episodes were performed for the purpose of pregnancy validation at the end of the 1st trimester equivalency of human pregnancy, prior to arrival of the animals into our lab (Figure 1). Maternal vital signs within these four groups showed different features under protracted anesthesia associated with the first infusion episode. Unique to the Control group, maternal heart rate increased while carbon dioxide significantly decreased over the course of anesthesia (Figure 2(a)). The increase in heart rate has been reported during anesthesia and is attributed to a decreased vagal stimulation [44]. The absence of such effect in the remaining three groups under our analysis indicates the physiological consequences of a prior anesthesia episode or alcohol intoxication on effects that are usually observed under anesthesia in the absence of these factors. One of the intriguing findings was the increase in maternal temperature throughout anesthesia in control groups, regardless of the presence or absence of a prior anesthetic episode (Figures 2(a) and 3(a)). This finding was surprising, because general anesthesia is known to impair thermoregulation, and thus to trigger hypothermia in humans and nonhuman primates [45, 46, 47]. However, the increase in temperature that we observed was not dramatic and reached +0.83 °C at its maximum. Moreover, it was observed at the end of the 100–200 min time interval from the beginning of anesthesia. Thus, we speculate that a modest temperature increase in baboons may represent an adaptive change to anesthesia-driven hypothermia and/or an adaptive response to physiological stress. Apparently, such responses are not observed in humans, as the incidence of hypothermia increases with longer anesthesia episodes [47]. The time-dependent increase in temperature in our analysis of baboon anesthesia records was absent in both alcohol-exposed groups (Figures 4(a) and 5(a)). This outcome is somewhat expected, considering the widely reported poikilothermic effect of alcohol leading to heat loss and reduction in body temperature [48, 49].

Unlike temperature, the increase in maternal oxygen level throughout anesthesia was common between the Control-PA and Alcohol-PA groups (Figures 3(a) and 5(a)). This commonality was quite unexpected, as the pulse oximetry approach utilized for peripheral capillary oxygenation measurement seems to be sensitive to an alcohol-induced effect on microcirculation. However, the finding implies the ability of a prior anesthetic episode to provide the basis for a time-dependent rise in pulse oximetry readings during prolonged anesthesia.

Overall, vital signs in the four groups under analysis exhibited features that were unique to a particular group. Notably, isoflurane level was maintained at a relatively constant level throughout the infusion in all groups. Thus, observed changes in maternal parameters were not caused by the differences in isoflurane level administered to the test animals. Interestingly, none of the vital signs exhibited time-dependent changes that would be common between both alcohol-exposed groups (without and with prior anesthesia: Figures 4(a) and 5(a), resp.). This finding suggests a critical contribution of a prior anesthetic episode to the effect of alcohol under anesthesia on maternal vital signs.

This conclusion was further bolstered by the comparison of average maternal vital signs between the groups (Table 1). Dams in the Alcohol-PA group rendered significantly lower (~–4%) oxygen (measured by pulse oximetry as peripheral capillary oxygen saturation) when compared to the Control group. This drop was accompanied by a nearly-significant decrease in systolic blood pressure when compared to the Control group. However, blood pressure parameters and respiratory rates were nearly identical in Alcohol-PA and Control-PA groups. Yet, the former presented a drop in peripheral capillary oxygen saturation. Although statistically significant, a 4% change in peripheral capillary oxygen saturation is not dramatic and falls within widely reported differences between pulse oximetry readings and arterial blood oxygen saturation [50]. Pulse oximetry readings do not always reflect arterial blood oxygen saturation, especially in vulnerable patient populations (such as critically ill adults or preterm infants); see [51, 52]. Thus, the observed drop in peripheral capillary oxygen saturation is not necessary expected to correlate with changes in endtidal carbon dioxide or systemic hemodynamic parameters. Notably, a discrepancy between pulse oximetry readings and arterial oxygen saturation becomes more apparent when microcirculation is obstructed, such as during vaso-occlusive crisis [53]. Considering that alcohol is known for its modulatory action on peripheral microcirculation [54], detected changes in peripheral capillary oxygen saturation in the Alcohol-PA group may represent a characteristic performance of pulse oximetry under alcohol-driven modification of microvascular function.

Regardless the underlying cause, changes observed in the Alcohol-PA group were not detected in either of the remaining groups (Control-PA and Alcohol). The unique vital sign profile of dams that were subjected to a previous anesthetic episode during pregnancy is alarming, as it suggests the ability of a short anesthetic episode in early pregnancy to define maternal vital signs during a subsequent exposure to alcohol and anesthesia. Our finding complements earlier reports that described the detrimental effect of anesthesia during the 1st trimester on pregnancy outcomes [11]. In our analysis, an episode of prior anesthesia was achieved with ketamine. Aside from its use in surgical practice, ketamine is often abused as a psychoactive substance, including consumption by pregnant women [55]. Besides the fact that repeated ketamine use and/or abuse results in fetal growth restriction and several characteristics of neurological abnormality, a brief intervention with ketamine is able to induce labor [56].

The Alcohol-PA group exhibited 50% fetal loss—the largest rate of negative pregnancy outcome in our analysis (Figure 6). Considering the significantly lower maternal oxygen in this group when compared to the Control group (Table 1), the high fetal loss is not surprising. Indeed, hypoxia has been recognized as a contributor to fetal growth restriction and pregnancy loss [57, 58, 59].

One of the objectives of our retrospective analysis was to establish whether any of the commonly monitored maternal vital signs under anesthesia could be predictive of fetal loss. Currently, the identification of reliable predictors of negative pregnancy outcomes represents a topic of active investigation. Detection of pregnancy risk as early as possible would lead to therapeutic interventions to potentially prevent fetal loss. In humans, such prediction of fetal loss in the first half of pregnancy can be achieved by the analysis of several factors which range from demographic and socioeconomic parameters to maternal health status and early fetal development parameters. For instance, advanced maternal age, history of vaginal bleeding, low fetal heart rate, low values for gestational sac diameter, and high yolk sac diameter, in various combinations, have been reported as predictors of pregnancy loss [60, 61, 62]. Consistent with the complex interplay of factors that orchestrate pregnancy loss, our study did not detect a single parameter within the maternal vital signs observed that could be predictive of impending pregnancy loss.

We recognize that the combination of alcohol with anesthesia during pregnancy represents a highly rare case, most likely driven by an emergency. In this situation, the great risk that is posed by anesthesia (Figures 5 and 6) merits investigation. Clearly, prior health history of the patient (i.e., presence of a prior anesthetic event and/or history of ketamine use) should be given careful consideration when weighing the risks of additional anesthetic intervention. Our findings may also be used by investigators and veterinarians in the field of alcohol research who utilize animal models. Last but not least, insights into the negative effects of combining anesthesia with alcohol on maternal vital signs and pregnancy outcome may contribute to our understanding of possible mechanisms that drive the interaction of alcohol with anesthesia.

Overall, our data highlight the disruptive nature of combining anesthesia and alcohol on maternal vital parameters and pregnancy outcomes. These findings are cautionary regarding the use of anesthetics in combination with alcohol during pregnancy.

## Figures and Tables

**Table 1:** Results of the linear regression model comparing each group (Beta ± SE, P-value) against Control. Parameter estimate beta represents mean difference in the outcome measure relative to the Control group. SE: standard error.

Parameter	Control	Control-PA	Alcohol	Alcohol-PA[.75pt]
Isoflurane (%)	1.70 ± 0.12	−0.05 ± 0.20, *P* = .8097	−0.06 ± 0.17, *P* = .7332	0.025 ± 0.20, *P* = .8990
Heart rate (beats/min)	101.00 ± 4.74	−4.99 ± 8.00, *P* = .5446	−11.59 ± 6.81, *P* = .1144	−3.73 ± 7.72, *P* = .6379
Respiratory rate (breaths/min)	18.60 ± 3.00	−8.55 ± 5.09, *P* = .1191	−2.59 ± 4.33, *P* = .5638	−8.02 ± 4.95, *P* = .1313
Temperature (°C)	36.60 ± 0.23	−0.41 ± 0.38, *P* = .3018	0.13 ± 0.33, *P* = .6879	0.06 ± 0.37, *P* = .8826
Oxygen (%)	97.20 ± 1.05	0.17 ± 1.77, *P* = .9236	−1.14 ± 1.51, *P* = .4667	−4.04 ± 1.71, *P* = .0359*
Carbon dioxide (mmHg)	45.60 ± 3.03	−15.45 ± 5.11, *P* = .0106*	−9.51 ± −9.51, *P* = .0495*	−14.62 ± 4.95, *P* = .0120*
Systolic blood pressure (mmHg)	78.40 ± 6.07	−22.43 ± 13.95, *P* = .1362	−1.71 ± 8.97, *P* = .8520	−21.22 ± 9.78, *P* = .0527
Diastolic blood pressure (mmHg)	30.60 ± 2.63	−4.99 ± 6.31, *P* = .4456	3.52 ± 3.91, *P* = .3873	−3.33 ± 4.18, *P* = .4423

* *P* < .05. Differences that are unique to Alcohol-PA group are underlined.

## Abbreviations

BAC: blood alcohol concentration
dGA: days of gestational age
